# Low-Grade Myofibroblastic Sarcoma of the tongue: A case report and literature review

**DOI:** 10.4317/jced.63065

**Published:** 2025-10-01

**Authors:** Evangelos Kalfarentzos, Venetia Louka, Charalampos Gkilas, Nikolaos Katsoulas, Penelope Korkolopoulou, Nikolaos Kolomvos

**Affiliations:** 1 DDS, MD, PhD, Assistant Professor OMFS, University of Athens Dental School, Department of Oral and Maxillofacial Surgery, “Evaggelismos” General Hospital, Athens, Greece; 2 DDS, Associate, School of Dentistry, National and Kapodistrian University of Athens, Greece; 3 DDS, MSc, Private Dental Practice, Athens, Greece; 4 DDS, MD, MSc, Pathologist, 1st Department of Pathology, School of Medicine, National and Kapodistrian University of Athens, Greece; 5 MD, PhD, Professor, 1st Department of Pathology, School of Medicine, National and Kapodistrian University of Athens, Greece; 6 DDS, MSc, PhD, MD, Assistant Professor, Department of Oral and Maxillofacial Surgery, School of Dentistry, National and Kapodistrian University of Athens, Greece

## Abstract

Low-grade myofibroblastic sarcoma (LGMS) is an extremely rare mesenchymal neoplasm. It arises from the differentiation of myofibroblasts and demonstrates a preference for the head and neck region. Herein, we present a case of LGMS in a 52-year-old man with painless swelling in the anterior right area of the tongue. Histologically, the lesion consisted of spindle-shaped cells exhibiting mild to moderate nuclear atypia, arranged in fascicles with a storiform pattern. Neoplastic cells were positive for SMA and negative for EMA, CD34, h-caldesmon, desmin, β-catenin, S100, SOX10, and NF, with a Ki-67 index of 15-20%. Complete surgical excision with clear margins was the treatment of choice. Very few cases of LGMS have been documented in the literature, and regular follow-up for these patients is essential for drawing reliable conclusions regarding local recurrence and the metastatic potential of this tumor.

** Key words:**Low-grade, myofibroblastic, sarcoma, tongue, myofibroblasts, immunohistochemistry.

## Introduction

Low-grade myofibroblastic sarcoma (LGMS) is a rare malignant tumor of mesenchymal origin and unknown etiology. It originates from the differentiation of myofibroblasts and is characterized by heterogeneity in histologic patterns, which, in some cases, makes the diagnosis problematic. Mentzel *et al*. initially reported it as a distinct entity in 1998 [1]. To date, only a few cases of LGMS in the Oral and Maxillofacial region (OMR) have been reported in the literature.

LGMS primarily affects adults, with a slight male predominance; a limited number of cases have been reported in children. The head and neck region is the most common location, with a predilection for the oral cavity, particularly the tongue, followed by the gingiva, mandible, and maxilla. Cases have also been described in the trunk, extremities, and the abdominal and pelvic cavities. Patients present with a painless, slowly enlarging mass or swelling, with its size varying from 1.4 cm to 17 cm [1].

This study presents a case of LGMS of the tongue in a 52-year-old male smoker with a non-remarkable medical history.

## Case Report

A 52-year-old male presented complaining of a painless swelling in the right anterior region of the dorsal surface of the tongue, which had been noted for the past month. Except for tobacco abuse, no significant medical history was appreciated.

During the clinical examination, no visible lesions were observed on inspection of the tongue. However, a well-circumscribed oval non-mobile nodule measuring 1x0.6x0.5 cm was detected on palpation. Antibiotic treatment with Amoxicillin/Clavulanic Acid 825/125mg for six days and re-evaluation were recommended to exclude a foreign body reaction. Upon re-examination, the lesion was found to be stable, and therefore, an incisional biopsy was performed.

**Figure 1:**
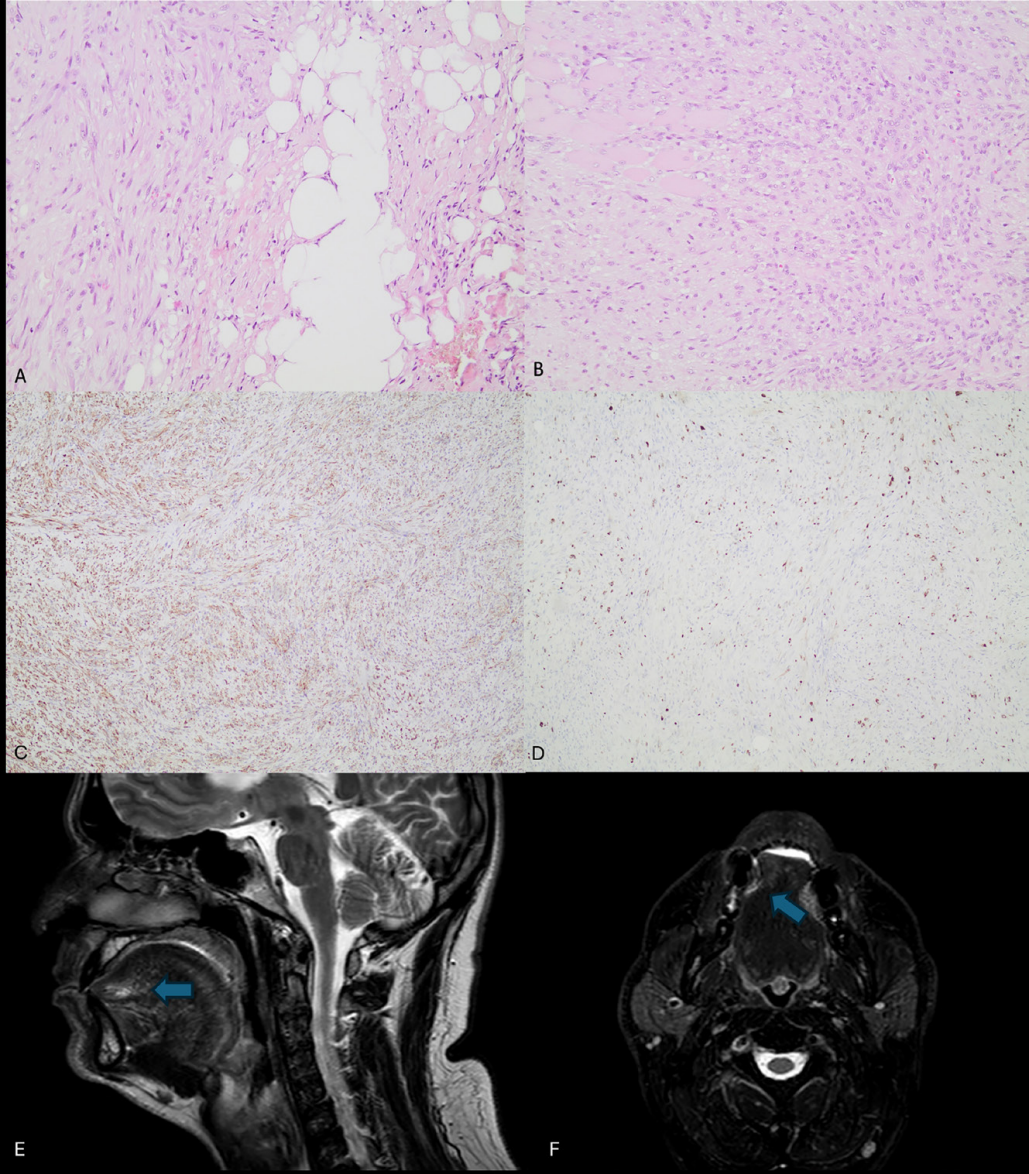
(A, B) Spindle cell proliferation with low-to-moderate nuclear atypia, infiltrating adipose tissue (A, HE X200), and muscle fibers (B, HE X200). (C) The neoplastic cells show diffuse positivity for SMA (X100). (D) The Ki-67 proliferative index is approximately 15-20% with heterogeneous expression (X100). (E, F) Magnetic Resonance Imaging of the head and neck region. The lesion is featured with an increase in T2 signal.

Histopathological evaluation showed a diffusely infiltrative soft tissue neoplasm composed of spindle-shaped cells. The cellular aggregates were arranged in fascicles that showed an infiltrative pattern among muscle fibers. Low-to-moderate nuclear pleomorphism and atypia were noted, along with partially increased mitotic activity (6-7 mitoses/10HPFs). The stroma exhibited myxoid areas and increased vascularity. Further immunohistochemical evaluation was performed, which showed positivity for SMA, while EMA, CD34, h-caldesmon, desmin, S100, SOX10, and NF were negative. No aberrant nuclear expression of β-catenin was observed. The Ki-67 proliferation index ranged from 15% to 20% with heterogeneous expression. Based on the above microscopic findings, LGMS was rendered as the final diagnosis (Fig. 1A-D).

Magnetic resonance imaging (MRI) of the craniofacial region and thoracic computed tomography (CT) were performed for tumor staging. MRI revealed features suggestive of a lesion, which were non-diagnostic due to changes induced by the prior biopsy. On imaging, no lymph node enlargement was detected (Fig. 1E,F).

**Figure 2:**
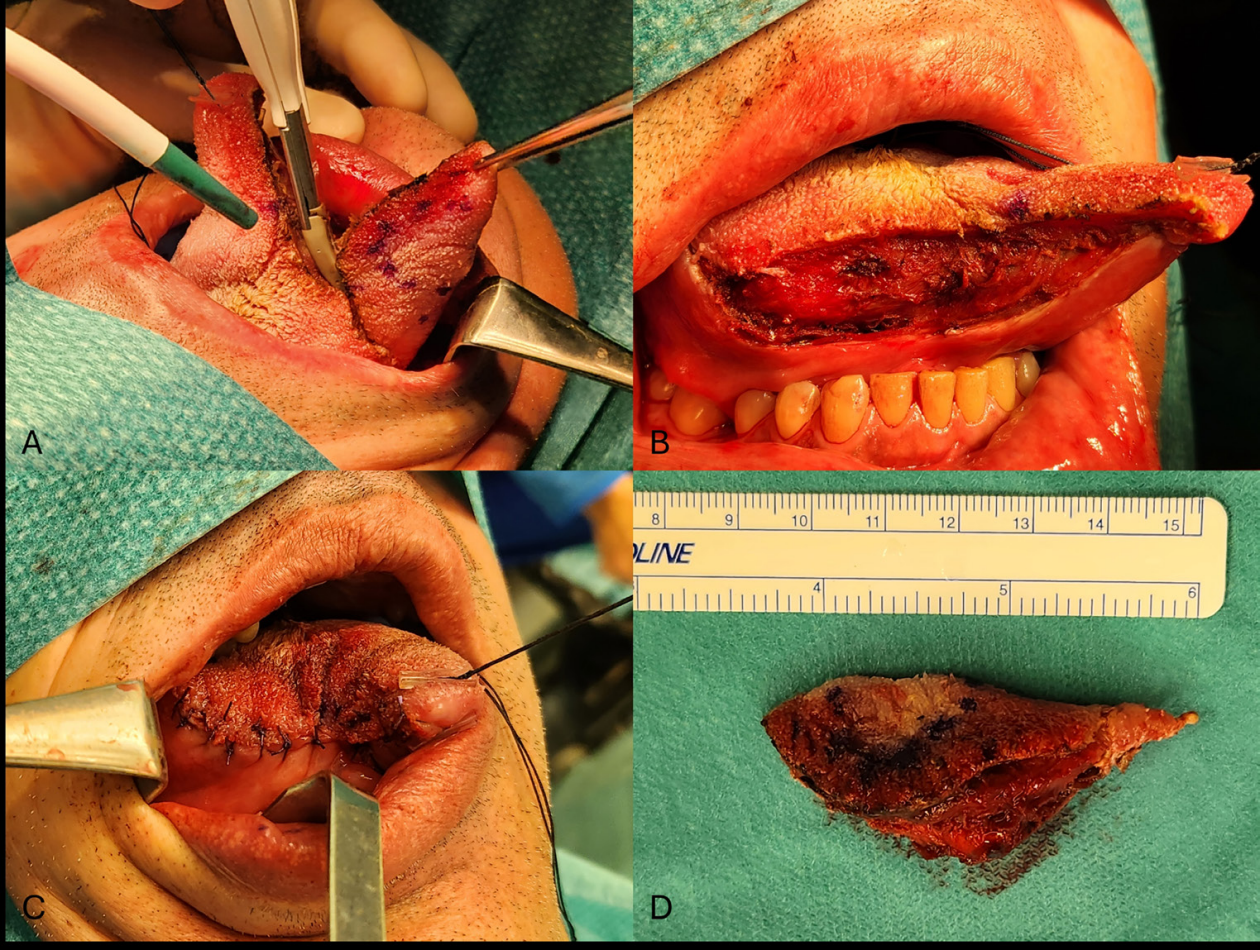
(A) The patient underwent a partial glossectomy. Tumor excision was performed using an ultrasonic scissors scalpel with a 1.5 cm margin of clearance. The lesion is indicated at the center of the segment. (B) The tongue, after tumor excision. No bleeding is observed when the ultrasonic scissors scalpel is used. (C) The tongue after suturing. (D) The soft tissue specimen postoperatively. The tongue segment dimensions were 4.5 x 2.5 x 2.2 cm. It was sent to the lab for histopathological analysis.

The patient underwent a partial glossectomy with clear margins using an ultrasonic scissor scalpel (Fig. 2A-C). During the second surgery, a distinct sclerotic area was detected that could not be considered a remnant from the former operation or fibrous connective tissue. Intraoperative frozen sections confirmed the tumor-free margins.

The excised specimen measured 4.5 x 2.5 x 2.2 cm (Fig. 2D) and contained an intramuscular, whitish nodule, 0.5 cm in its most significant dimension. Further histopathological and immunohistochemical evaluation confirmed the same microscopic findings as stated above, resulting in a diagnosis of LGMS, grade 1, according to the FNCLCC classification system, with clear surgical margins.

**Figure 3:**
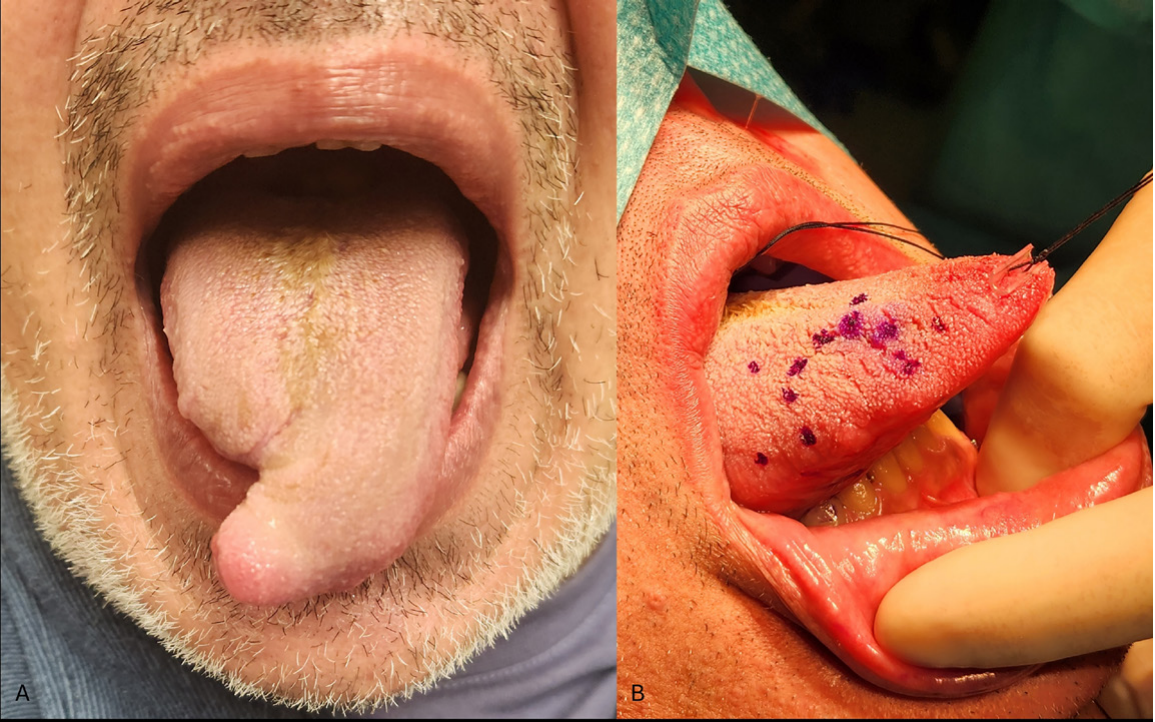
(A) Fourteen months postoperatively, the patient is alive and functional, with satisfactory speech. No local recurrences or distant metastases have been observed. (B) The tongue preoperatively. The outer points indicate the excision margin (1.5 cm), and the inner ones surround the lesion.

The patient has been on a regular follow-up program. Clinical examinations and magnetic resonance imaging (MRI) of the craniofacial region are performed every three months, and computed tomography (CT) scans of the thorax are performed every six months. Until now, the patient has remained disease-free, with no local recurrence or distant metastases observed sixteen months after the operation. Due to the preservation of the tip of the tongue, his speech ability was satisfactory postoperatively and further enhanced after speech therapy sessions (Fig. 3A). Since no metastasis was detected during the imaging examination, lymph node dissection was not required.

## Discussion

Few LGMS cases have been reported in the literature, with only 63 cases in the OMR, including the case described herein [2]. The rarity of the tumor and the lack of definitive diagnostic criteria make the diagnosis of LGMS even more challenging. The relatively indolent growth may contribute to a delayed diagnosis, particularly when the tumors are in deep-seated areas, such as the nasal and paranasal cavities [3].

LGMS primarily occurs in male adults, with an average age of 40 years [4]. Cases in infants, children, and adolescents have also been reported [5]. The head and neck region is more frequently affected, although it may arise in any soft tissue area, such as the abdomen, chest, and extremities. According to Lu Wang *et al*. the most frequent bone location is the distal femur [6]. As for the cervicofacial region, the most common sites are the tongue, the gingiva, the maxilla, and the mandible.

Histopathologically, the tumor consists of aggregates of spindled stromal cells that may exhibit a fibromatous or fibrosarcomatous growth pattern. No overt malignant cytologic features are noted; however, the tumor shows an infiltrative front in the adjacent soft tissues. High mitotic count, necrosis, or overt atypia are not typical features of the neoplasm, although rare cases of high-grade transformation have been reported [4-6,8]. The myofibroblastic nature of the tumor is highlighted by the immunohistochemical positivity of the neoplastic cells for smooth muscle actin (SMA), with varying degrees of desmin, calponin, and CD34 positivity. As such, the histopathologic differential diagnosis includes nodular fasciitis, inflammatory myofibroblastic tumor (IMT), solitary fibrous tumor (SFT), myofibroma, fibromatosis, fibrosarcoma, and leiomyosarcoma [4]. The use of the appropriate immunohistochemical panel and the pertinent molecular studies may aid in the final diagnosis.

The treatment of choice is complete surgical excision of the tumor with a margin resection of >2cm, to reduce the risk of relapse. Kim *et al*. suggest a standard resection of 3 cm [7]. However, this is almost impossible to achieve in areas such as the OMR and the neck, a fact that may contribute to a higher recurrence rate, up to one-third of cases, in this area [3,7]. In our case, we achieved 1.5 cm of tumor-free resection margins (Fig. 3B), which were confirmed by both the intraoperative frozen section biopsies and the histopathologic evaluation of the surgical specimen. Except for surgical treatment, adjuvant radiotherapy has been suggested with controversial outcomes, as some studies suggest that it may downgrade prognosis by even inducing metastatic potential. The role of adjuvant chemotherapy also remains unclear, although it appears to be of limited benefit. Some authors recommend this approach in cases where complete tumor excision is difficult, the tumor exhibits invasive features, and there is evidence of hematological or lymphatic metastasis. Y. Xu *et al*. in their 2020 study suggest that adjuvant chemotherapy and radiotherapy in patients with LGMS are not correlated with improved survival and should not be performed as part of routine treatment of LGMS [9-12]. In 2023, Bao Sun *et al*. presented the first case of immunotherapy treatment for LGMS in a 40-year-old male with a recurrent LGMS of the pharynx. The patient was initially treated surgically, but after more metastases occurred in the lymph nodes and the lungs, he underwent combined therapy with anlotinib and pembrolizumab for four cycles. He switched to monotherapy with pembrolizumab for 22 cycles because of the side effects of anlotinib, which resulted in a complete response of the patient and no detecTable signs of the disease. Despite the limitations of this report, it suggests that immunotherapy may be a significant treatment option for LGMS. However, further studies are needed to clarify the most effective treatment for LGMS. It appears that the selection of possible postoperative treatment may depend on the tumor’s invasion status and whether the tumor was excised entirely [13].

The life expectancy of patients with LGMS is still vague. A population-based study conducted in 2016 by Chan *et al*. found an overall 5-year survival of 71.6% in 49 patients [14]. Furthermore, the cohort study of Y. Xu *et al*. in 2020 showed that the average overall survival among 96 patients with LGMS was 125.2 months. In addition, it suggested that age at diagnosis is an independent prognostic factor, and the absence of surgical treatment is a poor prognostic factor for overall survival [8]. Chan *et al*. found that an age greater than 60 years was associated with worse overall survival and disease-specific survival [14].

Even though LGMS is classified as a low-grade tumor, it has a high tendency of local recurrence (24.59%) and a moderate metastatic potential (6.55%) [15]. Metastases have been reported in the lungs, heart, skull base, humerus, and other locations. Multiple metastases may occur in any of these body parts. In some cases, LGMS exhibited high-grade malignancy features, including ulceration and spontaneous necrosis [5,6,8].

In conclusion, LGMS is a rare solid infiltrative soft tissue and bone tumor of myofibroblastic origin with a predilection for the head and neck region. Its clinical and histological resemblance to benign lesions may lead to misdiagnosis and mistreatment. The preferred treatment approach is complete surgical tumor excision with clear margins. The resection width has been strongly associated with the possibility of local recurrence and metastasis, and it is generally accepted as a prognostic factor. Considering the scarcity of cases in the literature and the lack of diagnostic and treatment guidelines, strict and prolonged clinical surveillance of these patients is necessary.
